# Elements of Pathological Anatomy; Illustrated by Colored Engravings and 250 Woodcuts

**Published:** 1846-02

**Authors:** 


Art. IV.—*Elements of Pathological Anatomy;* Illustrated by colored engravings and Two Hundred and Fifty Woodcuts. By Samuel D. Gross, M.D., Professor of Surgery in the Medical Institute of Louisville; Late Professor of Pathological Anatomy in the Medical Department of the Cincinnati College; Surgeon to the Louisville Marine Hospital, etc., etc. *Second Edition, thoroughly Revised and greatly Enlarged.* Philadelphia: Ed. Barrington & Geo. D. Haswell. Louisville: James Maxwell, jr. 1845. Super-royal 8vo. pp. 822.

[continued from page 48.]

In our last number we followed the author through the chapters on inflammation and the effusion of serum; we proceed now to notice some of the remaining conditions and consequences of the inflammatory process.

Chapter IV.—*Lymphization.* The effusion of lymph, according to the author, like that of serum, is invariably of inflammatory origin; so invariably that "it may be assumed as a general proposition, than which there is none more satisfactorily established in pathological science." Except the followers of Macartney, few at the present day will deny this. Yet like as with serum, it is often the only symptom to be met with after death. In arachnitis large quantities are often poured out, though not the slightest redness, thickening, or opacity, can be discovered. Its appearance is not uniform, but may be modified by a variety of circumstances.

"In general, it is transparent, and of a white, yellowish-white, or opaline tint; but it may be opake, cineritious, milky-white, or reddish. In jaundice, I have seen it of a pale yellowish hue, from the presence of the coloring matter of the bile. At first it is very soft, semi-liquid, or almost diffluent, and so viscid that it may be drawn out into thin filaments; but it gradually increases in consistence, assumes a retiform arrangement, and feels very much like a mass of cobwebs moistened with water. When sqeezed, it yields a small quantity of fluid identical with the serum of the blood."—46.

Its microscopical characters as depicted by Gulliver, are given by the author, but as they are almost unintelligible without the accompanying cuts, we make but the following extract:

"Under the microscope, the mass of lymph is found to be composed of a great number of globules, connected together by a transparent matrix, and pervaded by exceedingly minute granules. The globules are of the same size as those of pus, and are at first very loose in their texture, but become gradually more dense and compact. In many cases, the substance consists of delicate fibrils, straight, transparent, running parallel with each other, and interspersed with small granules." —46.

Like other secrêtions, lymph is separated from the blood by a peculiar process; in chemical composition it is essentially the same wherever it occurs, and is similar to, if not identical with, the fibrin of the blood. It is now known that the quantity of fibrin in the blood is increased during inflammation, generally in direct proportion to the intensity of the morbid action.

Effused at first in globules, which become confluent, this substance assumes various forms, governed in some measure by that of the part producing it. In the mucous cavities, as the larynx, it sometimes moulds itself upon the part; in the peritoneum it is seen in bands and films; in the pleura it occurs in lamina or sheets; in the cellular tissue its shape is irreg ular. Likewise it varies in amount. The non-assimilative structures, as the serous sacs, appear to furnish it most abundantly. It is produced less abundantly and far less readily by the mucous membranes, though the mucous lining of the respiratory and intestinal tubes sometimes furnish it in large quantity. It is observed in the uterus and the œsophagus; it causes obliteration of the bronchia, fallopian tubes, and biliary ducts, stricture of the urethra, adhesion of the vagina, and closure of the lachrymal canal. It is furnished readily by the inner coat of the arteries, less by that of the veins; it is yielded sparingly by the skin and absorbents, the muscles and fibrous membranes, the tendons and ligaments, the cartilages and bones. Of the viscera, those furnish it most abundantly in which there is the greatest amount of loose cellular tissue.

The deposition of lymph takes place soonest when inflammation is most intense; it is also influenced, as we have just seen, by the nature of the affected part. The author has seen it covering the pleura and peritoneum nine hours after a gun-shot injury; Thomson observed it in the inferior animals four hours after the infliction of a wound; and in some cases it is thought to begin with the inflammation itself.

This effusion, wherever it occurs, may sooner or later become organized, endowed with bloodvessels, with absorbents, and by inference with nerves; may secrete, and absorb, and nourish itself; may, under the inflammatory state, pour out serum, tubercles, lymph, pus, and blood—in short, it may do all those acts and things which distinguish an original part of the living body.

This organization takes place most readily in connection with the serous sacs, the skin, cellular tissue and bones, and not at all, or only after long contact, in the mucous canals. It is not always the most intense inflammation which is most favorable to it, but rather the slow, moderate action. In the former case, it would seem that this substance is not well elaborated, or is furnished in such large quantity that a portion degenerates into pus. In certain inflammations, as erysipelas, lymph is readily effused, but it is ill-elaborated, of weak vital power, and the pus subsequently poured out is not walled about by it as in phlegmonous inflammation, but is diffused among the tissues causing an immense degree of mischief. The same thing occurs in connection with poisoned wounds, in many of which the exciting cause produces rapid deterioration of the blood, and consequently the lymph secreted from it is of a degenerate character. In other inflammations of a low form, occurring in certain states of constitution, attended by poor or watery blood, the organization of the effused matter is imperfect; it is liable to ulterior changes—contraction, shrinking—to softening, from inflammatory reaccession—and these changes are the source of injury to the surrounding parts.

The vascularization of lymph may take place in a few hours from the time of its commencement, or occupy several months. Home injected an artery and vein in some lymph, twenty-four hours after its effusion. The mode in which it is produced, has given rise to a good deal of discussion. According to one opinion, blood corpuscles escape from the adjacent original vessels, and after oscillating for a time in the effused lymph, shoot across and join the return veins; through the channel thus formed other corpuscles follow in a continuous stream, establishing its circulation; and from this first channel others are formed in a similar mode. The other opinion supposes the lymph to have a self-organizing power, by virtue of which it creates its own bloodvessels. We have already alluded to the microscopical characters of lymph when first effused. Its spontaneous coagulation is one step towards vascularization. In this state it presents numerous fibrils crossing each other in various ways, mixed with "exudation corpuscles" simple and compound—the former consisting of groups of granules and molecules; the latter, of cells with nuclei and nucleoli (granules and molecules). This is already a species of organization, and it may advance to a higher grade, or it may degenerate. When the former occurs, it is supposed (for the process is not exactly ascertained) that the compound exudation corpuscles (the cells with nucleoli) called nucleated cells, arrange themselves linearly, elongate, and form a communication by decadence of the opposing walls; in the channel thus formed the central or contained nuclei first oscillate, then are moved forward by the heart's impulse received through the neighboring vessels, and finally enter the general circulation. The nuclei are succeeded by blood corpuscles escaped from the adjoining capillaries, and thus, as before, canals are formed continuous with the original vessels. Others attribute to the lymph the power to form its own blood as well as bloodvessels, like the germinal vesicle of the embryo. The author gives the following account of the changes which take place whilst this self-organization is going on, without reference to the modern cell-theory:

"The primordial traces of the organizing process consist, according to the theory under consideration, in the appearance of a few red dots, which have been happily compared to the 'salient point' in the vitelline membrane of the chick, the fibrin being endowed, as was before stated, with a similar property of generating blood. Red furrows, streaks, or lines, are sometimes perceived, shooting out in various directions, which gradually assume the character of distinct vessels, in every respect analogous to those in other parts of the body. In other cases the small sanguineous trains take on a ramiform arrangement, and are ultimately converted into real vascular tubes. At an early period of their development, these trains are found, according to Laennec, to contain minute filaments of fibrin, permeable at their centre, which soon assume a cylindrical shape, and thus constitute the rudiments of the future vessels. These new channels, composed both of arteries and veins, the latter of which, however, predominate as well in volume as in number, gradually extend towards the neighboring parts, with the capillaries of which they finally inosculate, the blood passing freely from the one to the other. In their mode of arrangement, many of these vessels resemble the vena portæ; that is, they consist each of a single trunk, usually of considerable length, from each extremity of which are detached a certain number of branches, twigs, and filaments."—49.

Has this organized matter nerves and absorbents? The existence of both might be inferred from the fact already stated that false membranes perform the functions of secretion, absorption, and nutrition. The *absorbents*, however, have been demonstrated by Van der Kolk, who succeeded in injecting them with quicksilver.

The lymph thus organized forms the basis of the *analogous* tissues; and "by it wounds unite, bones knit, and arteries are consolidated." Upon its susceptibility of organization, and the capability of tissues to produce it, are founded many of the improvements of modern medicine and surgery. The tying of arteries for aneurism, the treatment of incised wounds, whether made by the surgeon or otherwise, and the different auto-plastic operations, are all based upon a knowledge of this wonderful power of organization. Indeed it may be said generally that when the practitioner knows the *habitudes* of the different tissues and organs in a state of inflammation, their liability to this or that effusion, whether serum, lymph, pus, or blood, he has gained a vast step in the way of prognosis and treatment. For example, if the surgeon have a wounded intestine he knows it will be useless to attempt a union of the two mucous surfaces; for morbid anatomy teaches the general truth, that mucous membranes are indisposed to the production of organizable lymph; but that between the two serous surfaces he may get a speedy and safe union. So with the physician: if he have to deal with an inflamed larynx, morbid anatomy teaches him that the danger consists in a closure of the glottis by an effusion of serum into the sub-mucous cellular tissue; if it be the trachea which is inflamed, that the danger arises from the effusion of lymph upon the mucous surface. We might bring forward other instances, but it would lead us too far, and besides they will suggest themselves to the mind of the reader.

A third product of inflammation, indicating a degree beyond that in which lymph is produced, is pus. In reference to the origin of this matter, the author says emphatically, that "*the formation of pus, in whatever part of the body occurring, is invariably the result of inflammatory action, either acute or chronic, simple or specific..*" Among the viscera, the lungs, liver, and kidney present pus most frequently; among the tissues, the cellular, cutaneous, mucous, and serous. All parts of the serous and mucous systems are not equally liable to it. As to the former, suppuration occurs most frequently in the pleura, the vaginal tunic of the testicle, and the serous linings of the larger joints. In the mucous system, it is much more frequent in the colon, than in the stomach or ileum, in the vagina than in the uterus, in the urethra than in the urinary bladder, in the nose than in the mouth, in the fauces than in the œsophagus, in the bronchiæ than in the larynx. The blood-vessels do not often suppurate, but the lymphatic system is frequently affected. Pus is deposited at first in globules; by softening and disintegration of intervening tissues, these become confluent, constituting abscesses; of these our author speaks of three kinds: the *acute*, the *chronic*, and the *metastatic*. The time required for the formation of an acute abscess depends upon a variety of circumstances; in the viscera from twelve to fifteen days; in the sub-cutaneous cellular tissue, less than a week, sometimes less than two days. Acute abscesses are rarely found in the lungs, brain, spinal cord, and spleen, death occurring before they have time to form. Situated externally, they tend toward the surface of the body; whereas visceral abscesses open into a splanchnic cavity, or into some hollow organ. The matter is often enclosed by a layer of organized lymph, called by Delpech the *pyogenic membrane*, varying in thickness from a fraction of a line to several lines, smooth and glistening, sometimes villous and granulated internally, and rough and flocculent externally.

In chronic abscesses the collection forms slowly, often unaccompanied by pain, heat, &c; whence the name *cold* sometimes applied to them. The cyst surrounding them, is of a dense fibrous or fibro-cartilaginous texture, often of considerable thickness. They are generally connected with the scrofulous diathesis, and occur most frequently in the sub-cutaneous cellular tissue, the lymphatic ganglions, and the dorso-lumbar portion of the spine. When the matter passes from one part to another it constitutes the *congestive abscess.* The *metastatic* variety affects most frequently the viscera, and of these, according to Dance, the lungs and liver, then the spleen, then the brain, heart, and kidneys. The number varies from one to many hundreds; the size—inverse of the number—varies from a hemp-seed to an orange. The contents of this abscess, especially when recent, are almost always semi-concrete, of a dirty greyish, or drab color; when of longer standing, they are more decidedly purulent. The surrounding parts may be healthy, but are generally altered in appearance, being engorged, discolored, softened, or converted into a pulpy substance; the coats of the vessels exhibit the marks of inflammation, in which the capillaries participate. These abscesses follow severe wounds, especially of the head; capital operations; compound dislocations and comminuted fractures; they occur also in the puerperal state, and after phlebitis, erysipelas, typhoid fever, &c. Their formation goes on stealthily, and generally requires from ten to fifteen days. "The most prominent symptoms are, violent rigors, usually paroxysmal in their character, delirium, stupor, prostration, and general insensibility." As to the cause of these collections, our author believes, with Cruveilhier and others, that they are the result of phlebitis, and that they may occur independently of suppuration in the organs which sustained the primary mischief.

With the appearance of pus—*healthy* or *laudable*, as it is called, as well as the *sanious*, the *fibrinous*, the *scrofulous*—the reader is doubtless familiar. The microscopical characters of pus are not unimportant. It consists of opake corpuscles suspended in a thin transparent liquor, mingled with more or less of the softened and disintegrated tissues of the part where it is formed. The characteristic pus globule consists of a spherical and externally rough envelope, composed of fibrin, containing two or three opake and exceedingly minute spherical granules, and some fluid. These globules are something larger than the red particles of the blood, and are evidently the exudation corpuscles, which, instead of undergoing further development, have degenerated. The author gives a full account of the analysis of pus by different individuals; from all which it seems to contain every element of the blood except the coloring matter. Purulent matter is frequently *contagious;* but to the eye in such cases it does not differ from that of common inflammation. Animalculæ have been observed in the matter of chancre, and in that from cancerous ulcer.

Pus, according to Gendrin and others, is sometimes an intra-vascular formation; if so, it must pass out of the vessels ere yet it takes on the globular form and while still granular, as the pus corpuscle cannot escape otherwise than by rupture of the vessel. It is sometimes absorbed; large abscesses are thus got rid of; in this case, again, the pus corpuscles must recede to the granular state, as, without rupture of a vessel, they can no more pass in than out. The fibrin of the pus thus absorbed seems to be unfitted for further use in the economy, and is eliminated through the kidneys.

*Hemorrhage,* which makes the subject of the fifth chapter, next claims our attention. Hemorrhage occurs under two very opposite conditions of the system: one a state of vascular repletion, or plethora, which it so often relieves; the other, and most frequent one, a state of anæmia, when blood is small in quantity and poor in quality. The morbid alteration of the blood which most frequently accompanies hemorrhagic effusions, is, according to Andral, diminution of the quantity of fibrin. Hemorrhage is most frequent in the mucous, cellular, and serous systems; and in the mucous system, is most frequent in the large bowel, then in the stomach, then in the ileum. Of the viscera, the brain and lungs are by far the most often affected. Hemorrhage occurring in the interior of a part is termed *extravasation.* The word *apoplexy*, formerly applied to effusions in the brain, is now used in reference to all extravasations.

When speaking of inflammation it was seen that a period came when the distended and softened coats of the vessels gave way, allowing the blood to escape in mass. Unquestionably rupture of the vessels is much the most common cause of hemorrhage, whether accompanied by inflammation or not. It is thought, however, that the blood may be secreted—exhaled—that is, pass out of the vessels by a sort of vital "endosmose, diapedesis, or transudation."

"Analogically," says the author, "it may be inferred that the blood vessels are in a state of morbid activity, whereby fluids are suffered to escape that were appointed to be retained; or it may be supposed, as we have reason to believe is often actually the case, that the capillaries, being in a state of debility and relaxation, have their pores rendered unnaturally patulous, and thus allow the blood to have a more ready egress."—64.

What sort of "morbid activity" this is, we do not exactly understand. Any activity of which we can conceive, would be directly opposed to transudation, whether vital or mechanical. That there is a loss of tone by the capillaries in many hemorrhages, we can readily understand. Still, as in the case of pus, in the present state of our knowledge, it is impossible to see how the unchanged blood particles can escape from the unruptured vessels. In the frog, Williams (*Outlines Path.*, Amer. Ed., p. 195) states that he has seen the red particles roll up like a rice wafer previous to passing through the walls of the vessels. Does an analogous change of form occur in human blood? Red particles are to be seen in the fluid poured out in many cases of hemorrhage; but may not these escape from rupture of some of the smaller vessels, whilst the great bulk of the so called blood consists of the liquor sanguinis and the coloring matter? But we will not pursue this subject any further.

The disposition to hemorrhage may be *hereditary.* Sometimes it affects all the male or female descendents for several generations; in other instances it disappears in one generation and re-appears in another, affecting indifferently both sexes. Hemorrhage is *vicarious.*

"As its name imports, it is supplemental of a similar natural or morbid state in a remote organ, and is most frequently observed in young females, in consequence of the tardy appearance of the menstrual flux, or from its suppression after it has been established. In the great majority of cases, it is located in the mucous membrane of the nose, and recurs with considerable regularity every lunar month, until the obstruction, of which it is the result, has subsided. Occasionally the blood oozes from the skin, the eye, ear, lung, anus, and even the nipple, either simultaneously or successively."—66.

Again there are *critical* effusions, as in protracted fevers, where vital power is lowered, and irregular accumulations of blood occur in certain organs.

When blood is effused it may be rejected, as when it occurs in the intestinal tube; or absorbed, as in the lungs and brain; or remain and become organized; or, lastly, act as a foreign substance, inducing fatal inflammation. As to the absorption of blood, the same difficulties occur as in the absorption of pus. The blood globules cannot pass unchanged into the vessels, any more than they can pass out, unless there be rupture of the parietes.

*Softening*—mollescence, ramollissement—the subject of the next chapter, is one of the most unequivocal signs of inflammation; occurs, though not with equal frequency, in nearly every organ in the body; and in some structures, the tendons and cartilages for example, is almost the only sign of inflammation. It is frequent in the brain and spinal cord, in the spleen, the liver, and the mucous membrane of the intestines.

It is common in the bones, and sometimes pervades the entire skeleton, constituting *osteo-malacia.* The inflammatory action accompanying it is generally acute, though sometimes it is of a mild or chronic grade. Rostan thought that in the brain it sometimes depends on ossification of the arteries; Bennett has shown that in these cases it is a modification of the process of suppuration. Softening would seem almost necessarily to precede suppuration and ulceration: the tissues are first filled with serum or the other fluid elements of the blood; nutrition is overborne, cohesion impaired; and molecular disintegration ensues, with discharge or absorption, or both.

A softened organ may be unnaturally white, as in the brain, or red, as in the lung (red hepatization), or any intermediate shade of color. The seat of this lesion seems to be for the most part in the cellular element, as might perhaps be inferred from its universality. It is also a post-mortem occurrence, as in the lung from gravitation or stagnation of the blood, and in the stomach from the action of the gastric juice.

Chapter VII. treats of *Gangrene*, which term the author uses as synonymous with mortification and sphacelus. We pass this chapter with a notice of one or two points.

Gangrene may arise from an impoverished condition of the blood. This is seen in the ill-fed, ill-clothed, and ill-housed children of the lower orders of society, in whom it attacks the groin, axilla, and feet, and the mucous membrane of the mouth and vulva. In France, Switzerland and Germany, the lesion is frequent, occurring in terrible and devastating epidemics, from the eating of grain affected with *ergot.* The parts which most frequently suffer are the extremities, generally the lower; it may involve a part or the whole of an extremity; is in most cases preceded by the local symptoms of inflammation; the parts are left unusually dry and hard, the gangrenous odor being generally absent. Similar effects have been produced by feeding spurred rye to some of the lower animals. The disease from this cause is rare in this country, especially in the valley of the Mississippi. It has prevailed at different times in Pennsylvania and New York among horned cattle; and in these instances, according to Mease, arose from the use of the *green grass*, the poa viridis of the botanists, the seeds of which were affected with ergot. Inflammation running rapidly into gangrene has been seen by Barbier and others to follow the inordinate action of emetic substances; the gangrene attacked the feet and toes, the hands, chin, lips, nose and ears; and seemed to arise from the sudden and great reduction of the powers of life.

*Senile* gangrene, occurring most frequently in the lower extremities, especially the feet and legs, is not confined, as the name imports, to the old. Of sixty-seven cases collected by Hecker, one occurred between the 1st and 10th year, six between the ages of 10 and 20, seven between 20 and 30, nine between 30 and 40. The largest proportion, however, occurred between the ages of 50 and 60, and 60 and 70, there being twelve in the former period, and nineteen in the latter. The progress of this affection is generally slow, and often unaccompanied by any local symptom except the discoloration. Pott, who first described it, thought it most frequent in males, in the proportion of twenty to one. In France the proportion is nearly the same in both sexes. In the cases just referred to, collected by Hecker, the males are to the females less than two to one. The author has seen but three cases, two of which were females. The cause is "undoubtedly inflammation of the lining membrane of the vessels, especially of the arteries, leading to an effusion of lymph, and the formation of coagula, fibrinous concretions, and other extraneous products in their interior." This may be so; we are disposed to think it is, in view of the dry, moistureless, and withered state of the affected part; but still may it not be the consequence of the disease, instead of its cause?

*Ulceration* is the subject of the eighth chapter. By this term the author understands "the solution of continuity of a texture from the absortion of its molecules." It is synonymous with the *erosion* of the older writers, and with the ulcerative absorption of Hunter and the modern pathologists. This lesion is by far the most common in the skin, mucous membranes, and cellular tissue. Next in order come the heterologous formations, the bones and teeth, the articular cartilages and their coverings. The proper serous membranes, the fibrous and muscular structures, rarely suffer; nor do any of the internal organs except the uterus. The blood-vessels have a remarkable exemption from this lesion; arteries are occasionally seen playing away whilst all around them is undergoing rapid destruction; the vessel in such cases is protected by a coating of lymph. The mucous membranes of the respiratory and the genito-urinary organs are rarely ulcerated. In the intestinal canal, the ileum and colon are much oftener affected than the stomach. Ulceration may be rapid or slow; destroying a large portion of one or several tissues in a few days; or enduring for months and years as in bone, skin, and mucous membranes. In its progress, it tends towards the nearest surface, or towards the termination of the arteries, as was mentioned in speaking of abscess.

The great cause of ulceration is inflammation conjoined with pressure. It may occur without pressure, as in the skin and mucous membranes; and again, pressure seems to be the chief cause, as in caries of bone from the presence of an aneurismal tumor. As predisposing to this lesion, may be mentioned that condition of the blood in which its solid or fibrinous elements are wanting, and in which, as we have already stated, irregular accumulations or congestions take place in various parts of the body. Vital power is lowered, and from trivial causes, from the application of blisters, from slight blows, inflammation is set up, and terminates in "a foul, sloughy, and irritable ulcer." Bed-sores of the hips and buttocks occur in low states of fever; old wounds break out afresh and fractures are disunited in scurvy; and in animals fed on non-azotised articles of food, ulceration attacks the cornea and other textures. The pain attending ulceration is sometimes inconsiderable; again it is "of a dull-aching or gnawing character, as if insects were feeding on the part." Sometimes it is constant, sometimes intermittent, sometimes periodical; and often is accompanied with hectic fever, rapid emaciation, and great reduction of the vital powers.

Ulceration is not always to be deprecated. It enables abscesses to *point;* it brings foreign bodies to the surface; and in old drunkards it forms a sort of safety valve by establishing sores on the extremities, thus saving them in all probability from internal disease.

We have quoted the author, and other writers, as saying that ulceration of a part depended upon absorption of its molecules. But is this true? We think not. Our limited space will not justify a discussion of the subject here, even if it were likely to be attended with any practical benefit. We cannot help remarking, however, that absorption is either diminished, or absent during the inflammatory process; that in the heterologous formations, ulceration readily occurs, absorption never; that previous to gangrene, arteries, veins, absorbents—everything is glued up by coagulable lymph, and that the dead part is pushed off as it were by pus, which flows away mingled more or less with the debris of the dead and dying tissues. In short, without denying that absorption sometimes exists, ulceration seems to us, in most cases, to be death by molecules, as sloughing and gangrene are death in mass.

Chapters IX and X.—We come next to *granulation* and *cicatrization.* Both are subjects of vast interest, but our notice of them must be brief.

Granulations are small, red, pointed, somewhat firm bodies, composed of coagulating lymph, which becomes organized in the manner previously mentioned. They are highly vascular; the vessels, which may be demonstrated by injection, are arranged in a tuft-like form, and those of adjacent granulations communicate only at the base. These little bodies are sensitive, sometimes extremely so, and hence have nerves; they are capable of absorbing various substances, and are themselves sometimes absorbed. They are developed most readily in parts that are lax and vascular. In mucous membranes, aponeuroses, ligaments, tendons, cartilages, and bones, they form tardily, and these are structures in which lymphization is imperfect. They are rarely seen in any of the viscera except the brain.

Cicatrization is the healing of wounds and ulcers, and, like resolution and gangrene, is a proper termination of the inflammatory process. All the textures of the body may be thus regenerated, except cartilages and muscles, and in one instance the author has seen muscular substance renewed. This regeneration, however, is never perfect. In cicatrix of the skin, for example, the inner surface of the new production is not reticulated, the mucous net-work is imperfect, there are no sebaceous follicles, nor follicles containing hairs. Cicatrices are liable to inflammation, and vital power being feeble, they are thus easily destroyed; to hypertrophy, and hence they have a rough knobby feel, and dense fibrous structure, creaking under the knife; to inordinate contraction, as after burns, producing horrible deformity, and after severe mercurial salivation, causing rigid and firm closure of the jaws; to semi-malignant growths, in the shape of a small dry excrescence covered with cuticle, soon becoming moist and partially ulcerated, secreting thin fetid and semi-purulent fluid, by and by converted into a more solid tumor—scirrhus or encephaloid—as large as a pullet's egg, followed by ulceration, fungous granulations, and death from accompanying discharge and irritation. After a cicatrix is removed, the second sometimes shows the same disposition to contract. Two years ago we saw a case of closure of the jaws from mercurial sloughing and ulceration of the gums and cheeks. The parts were perfectly rigid, the lower jaw scarcely moveable and firmly held against the upper by thick bands and sheets of fibro-cartilaginous structure. These were divided, with slight but temporary improvement. Next they were entirely removed, after which the jaw had considerable motion, and a machine was constructed so as to keep it constantly depressed. But it proved fruitless; for, as cicatrization went forward again, so did the gradual but firm contraction, rendering the counteracting force insupportable; and the little sufferer was sent away unrelieved. The malignant growth, just mentioned, rarely returns when removed.

The chapters that we have now noticed include the more direct and immediate conditions of inflammation. Before proceeding further it may not be amiss to remark that, although these conditions, or terminations as they are called, are thus isolated for the purpose of study, they do rarely, if ever, actually so occur in the living body. For example, the effusion of serum, except when it serves, like hemorrhage or bloodletting, to resolve or cut short an inflammation, is never seen alone, but exists throughout the whole process. In cicatrization, itself an absolute termination, there is an effusion of serum, of lymph, and of pus, not to mention organization, all going forward at once. Ulceration and suppuration co-exist with scarcely an exception, and both, as we have already intimated, are generally preceded by softening. Suppuration is seen in connection with lymphization, hemorrhage, and effusion of serum; as is fully recognised in the terms *fibrinous*, *sanguinolent*, and *serous*, applied to pus. Thus it is readily seen how complex a process is that of inflammation.

The succeeding chapters are devoted to some of the more remote consequences of inflammation—Induration, Hypertrophy, and Atrophy. By *Induration* the author means an increase of consistence in an organ, whether arising from deposition of new matter (excluding heterologous formations), from deficiency of natural secretion, or from transformation of some of its tissues. This lesion is an exceedingly common one, common to all the tissues in the body, though not equally so; occurs at any age, and even during intra-uterine life; exists alone, or associated with other alterations; and may affect a part or the whole of an organ, or only one of its anatomical elements. The *size* of a part indurated, like its *color*, may be natural, augmented, or diminished. An increase of size is most frequent, as in the liver, spleen, and lymphatic ganglions; so, too, the weight is more frequently above than below the natural standard. The *degree* of induration depends upon a number of circumstances.

In reference to the time required for the production of this disease, it may be said to be chronic, occupying weeks and months and years, as is far the most frequent; or acute, occupying but a few days. The chief cause of this lesion, is a deposition of coagulable lymph as a consequence of inflammation. Along with this there is frequently poured out, as in the lungs, more or less of the coloring matter of the blood, or the blood itself, giving the affected structure a red color. In hardening of the cellular tissue in infants, the coloring principles are two: one an orange red, the other bluish. Induration from deficiency of the natural secretion, is seen in the general drying up of the organs and tissues in old people; being most considerable in the cellular tissue, mammæ, ovaries, prostate gland, the voluntary muscles, and the bones. Induration from transformation, or the disappearance of some of the elements of an organ, is occasionally seen in the liver, spleen, and lungs, around hydatids, serous cysts, tubercles, &c. The lung affords an example when the copious effusion in chronic pleuritis reduces it to a mere cake, *ex*pressing its fluid contents, and *com*pressing its solid elements. Induration from this last cause is constantly associated with atrophy.

Induration when suffered to continue may lead to the cartilaginous, the fibro-cartilaginous, and osseous transformations. Of the two forms, the acute and the chronic, the latter is most amenable to treatment; which is something directed to the removal of morbid deposits—iodine and kindred articles internally, stimulating frictions externally—and must be long continued.

*Hypertrophy*, frequently accompanied by the last affection, is not so widely diffused, as no one has ever seen it in the serous membranes, ligaments, tendons, or fibrous envelopes. There are few, however, who have not observed it in the adipous tissue, heart, spleen, thyroid body, &c. &c. Like induration, too, it occurs at all ages, and, in the case of the thymus gland, possibly during uterine life. Hypertrophy may be general, occurring (with the restrictions just mentioned) in nearly every organ and tissue of the body at the same time; or local, occupying a single organ, or, w hatis more frequent, a part of an organ. The causes may likewise be general and local. Of the former we know but little. Among the local causes chronic inflammation holds an important place. It produces enlargement of the spleen, the liver, and the lymphatic ganglions, developes the follicles and villosities of the mucous coat of the intestines, and thickens the mucous tunic of the bladder. Increased action of an organ, to overcome some mechanical impediment to the performance of its function, is another cause: as in the heart, from hindrance to the exit of blood; in the muscular coat of the stomach, from obstruction at the pylorus; in the urinary bladder, from stricture of the urethra, or enlargement of the prostate. Hypertrophy also follows increased action in the discharge of healthy function. This is seen in the lung, the kidney, and the muscles of voluntary life. It is simply the physiological law upon which the physician acts when he directs, "plenty of exercise and plenty of fresh air," to give vigor and power to the enervated frame. An enlarged organ may have its consistence increased (induration), diminished (softening), or unaltered; the former is much the most frequent. Increase of weight is generally present, unless there be at the same time atrophy of some other element. Increased volume is by no means constant. Change of form occurs when the lesion is partial.

*Atrophy*, although existing sometimes in connection with hypertrophy, is a totally opposite lesion. Hypertrophy consists in an exaltation of the nutritive function, atrophy in its imperfect exercise. In the one, the body will attain the weight of seven hundred pounds, without an unusual quantity of aliment; in the other, it will fall to fifty-eight, with the usual quantity of food. General atrophy (marasmus, emaciation, consumption) attends divers chronic diseases of the lungs, heart, and stomach, It also occurs in the progress of age—*senile* atrophy.

"All animals have a period of growth, maturation, and decay. In the human subject, the body, after having reached the age of forty, begins to exhibit traces of decline, which from this time on become gradually more and more conspicuous, until the machine is literally worn out, and man 'goeth to his long home.' Examined at this period, the whole mass of the brain is generally found diminished in size, the nerves have lost their moisture, and the ganglia connected with them are condensed, and considerably shrunk in volume. The respiratory system experiences similar changes: the lungs are dryish, inelastic, and increpitous, their volume is sensibly lessened, the walls of the air-cells are attenuated, and whole lobules are sometimes deprived of their vesicular structure. The muscles of voluntary life are pale, flabby, and diminished in bulk; the arteries, veins, and absorbents shrink in their diameter, and a large proportion of the more minute ones, becoming useless, are obliterated, and lost; the lymphatic ganglions are hard, small, and many of them entirely dispapear; the bones are spongy, brittle, and extremely prone to fracture; the ligaments are unusually slender; the articular cartilages dry, and attenuated; and the salivary glands, together with the liver, pancreas, spleen, and kidneys, are indurated, and considerably reduced in size. In the male sex, after the functions of the testicles have ceased, absorption frequently commences in these bodies, which shrink, become soft, pulpy, and are sometimes not larger than a French bean. The cells of the penis are augmented, and their fibrous parietes very much attenuated, in some iustances even partially absorbed. In the female, the ovaries are pale, shrivelled, and frequently transformed into a condensed greyish substance; the mammæ are soft and flabby, with scarcely a trace of their original structure; and the uterus is hard, firm, and diminished in volume."—89, 90.

Local atrophy may affect the whole or a part of an organ, or one of its constituent elements; may exist singly, or in conjunction with hypertrophy, induration, or softening. The causes are: the cessation of function in an organ, as in the pupillary membrane, and wolffian bodies before birth, the ovaries and thymus gland after birth; diminution of nervous influence, as in the arm and leg from pressure on the axillary and sciatic plexuses; deficient supply of blood, as in the testicle when the spermatic artery is tied; inflammation, as in the gall bladder from the presence of biliary calculi, and in a thousand other cases.

The subject of the next chapter is one not found in the former edition, nor is it noticed, except incidentally, in most of the treatises on morbid anatomy.

*A Fistule*—fistula, as it is generally termed in the books—is always a consecutive disease, is developed by various causes, and may be formed wherever there are two contiguous cavities, or a cavity and an external surface. It has usually two openings, and then is said to be *complete;* when there is but one opening, it is termed a *partial* or *blind* fistula. Fistules receive various epithets according to their situation; as anal, recto-vaginal, vesico-vaginal, perineal, broncho-pleural, &c. They vary in length from a few lines to several inches; the largest usually observed in connection with psoas abscess, between the kidney and lung, and between different coils of intestine. The diameter is sometimes exceedingly small, scarcely admitting a bristle, as in lachrymal fistula; or so large as to admit the finger. The track may be straight, or exceedingly tortuous and winding; may communicate externally by one or several openings; and may have branches ramifying from it. A recent fistule is nothing more than a granulating surface; by-and-by it becomes lined by coagulable lymph, a true pyogenic membrane, of variable thickness—at first more or less red, afterwards white, grey, or bluish—smooth upon its free surface, or mammillated and villous—and covered by a thin glairy fluid, which is more or less mingled with the secretion of the part upon which the fistule opens. This lining membrane, in old cases, often acquires the consistence of fibro-cartilage; and the surrounding parts are likewise rendered firm and callous by coagulable lymph.

"There is a variety or form of fistule which may be regarded as a remnant of embryotic organization. Its most frequent situation is the antero-lateral part of the neck. Like the ordinary fistule, it may terminate in a cul-de-sac, or it may have two orifices, of which the external is sometimes scarcely visible. The abnormal passage itself is usually very narrow, and seldom extends beyond two or three lines in depth."—94.

We come next to *Transformations*, which means "those changes which a pre-existing tissue undergoes, as it is being converted into another that is totally different from it, but which has its analogue in the animal economy." Those admitted by the author are the cellular, mucous, cutaneous, fibrous, cartilaginous, osseous, and adipous. We shall have an opportunity of noticing these in connection with the different organs and tissues, and shall therefore pass them by now, with the remark that they are most frequent in old age, and in the opinion of the author are invariably dependent upon inflammatory irritation.

Chapter XVI.—*Pneumatosis, or collections of aëriform fluids.* Examples of this affection are familiar to all, in pneumo-thorax, emphysema, tympanitis, &c. The quantity of aëriform fluid is subject to great diversity: it may occupy the entire body, as in emphysema; or be restricted to a few lines, as in the lung; it may puff up the intestines, paralysing their muscular coat, displacing the abdominal organs, and interfering with the descent of the diaphraghan; or enlarge the uterus so as to simulate pregnancy. The *physical* and *chemical qualities* of the fluid are subject to equal diversity. In the lung and sub-cutaneous cellular tissue it generally occurs after wounds, and then resembles in all respects the atmosphere whence it is derived. In other situations it contains variable quantities of oxygen, carbon, hydrogen and nitrogen—sometimes it is inflammable from the large admixture of hydrogen, sometimes the reverse from the predominance of carbonic acid gas. In the alimentary canal, uterus, pleura, and in scrofulous abscesses, it is mingled with a greater or less quantity of sulphuretted hydrogen.

Air may find admittance through a wound, as in emphysema of the lung and sub-cutaneous cellular tissue; or through some natural opening, as when it is swallowed, or when it collects in the vagina and uterus from relaxation of the former organ. Again it may arise from the operation of chemical agents; as in the uterus, from retention of blighted ovum, portions of placenta, or of fœtal membranes, which undergo decomposition; in the intestinal tube, from the play of chemical affinities among the heterogeneous articles of food, especially when digestion is disordered; in the chest, when there is a collection of sero-purulent fluid; and in hernia, when sphecalus occurs. Lastly, it may result from a true vital process of exhalation or secretion. Analogical evidence is furnished by the fact that exhalation of oxygen and absorption of carbonic acid is constantly taking place in vegetables, that just the reverse process is going on with equal constancy in the human lung, and that the air in the air-bag of fishes is evidently secreted. It is ascertained also that an aëriform fluid exists in the blood, consisting, according to Krimer, of oxygen, carbonic acid, and hydrogen. Extrication of gas in different parts of the body has been observed to follow excessive losses of blood.

Spontaneous pneumatosis is most frequent in females about the decline of the menses. The development may be rapid; or gradual—some cases of physometra lasting for months and years. Sometimes it recurs periodically, every day, or every other day; it is also supposed that it may be hereditary, but the evidence is not conclusive. C.

[TO BE CONTINUED.]

